# Environmental DNA analysis for macro-organisms: species distribution and more

**DOI:** 10.1093/dnares/dsac018

**Published:** 2022-06-02

**Authors:** Toshifumi Minamoto

**Affiliations:** Graduate School of Human Development and Environment, Kobe University, Kobe, Hyogo 657-8501, Japan

**Keywords:** environmental DNA (eDNA), environmental RNA (eRNA), eDNA barcoding, eDNA metabarcoding

## Abstract

In an era of severe biodiversity loss, biological monitoring is becoming increasingly essential. The analysis of environmental DNA (eDNA) has emerged as a new approach that could revolutionize the biological monitoring of aquatic ecosystems. Over the past decade, macro-organismal eDNA analysis has undergone significant developments and is rapidly becoming established as the golden standard for non-destructive and non-invasive biological monitoring. In this review, I summarize the development of macro-organismal eDNA analysis to date and the techniques used in this field. I also discuss the future perspective of these analytical methods in combination with sophisticated analytical techniques for DNA research developed in the fields of molecular biology and molecular genetics, including genomics, epigenomics, and single-cell technologies. eDNA analysis, which to date has been used primarily for determining the distribution of organisms, is expected to develop into a tool for elucidating the physiological state and behaviour of organisms. The fusion of microbiology and macrobiology through an amalgamation of these technologies is anticipated to lead to the future development of an integrated biology.

## Introduction

One of the most concerning global environmental issues confronting society today is the loss of biodiversity.^1^ The numbers of wildlife species and individuals continue to decline significantly on a global scale, as emphasized by the current IUCN Red List (https://www.iucnredlist.org/), which indicates that approximately one-third of the assessed species are threatened with extinction. Populations are similarly undergoing marked declines, with losses in freshwater environments being considered the most serious.^2^ Such erosion of biodiversity will lead not only to the loss of ecosystem functions and the degradation of ecosystem services but may eventually contribute to the collapse of entire ecosystems on a global scale. Consequently, the conservation of ecosystems and biodiversity is an urgent issue for all societies, for which we need to develop an adaptive management approach.^3^ Essentially, this means that having implemented conservation measures, their efficacy should be closely assessed, and the information thus garnered applied in subsequent efforts. For this purpose, monitoring is important to determine the presence, distribution, and numbers of any given species.

Traditionally, the monitoring of organisms has involved the collection or observation of individuals and visual morphology-based identification. For the collection of aquatic species, methods such as fishing, electro-fishing, and the use of cast nets, gillnets, and trawls are routinely employed, whereas scuba diving and, more recently, underwater drones are used for visual observations. However, all these methods have the notable disadvantages of being labour-intensive and costly. Consequently, the monitoring of aquatic organisms to date has been far from comprehensive, whereas the loss of biodiversity continues at an unprecedented rate.

## Determining the distribution of macro-organisms using environmental DNA

In recent years, there has been a rapid development of environmental DNA (eDNA)-based analyses, in which information obtained by sampling DNA from environmental sources is used to identify the distribution and abundance of macro-organisms such as fish and amphibians. In this context, eDNA refers to all DNA in the environment, which, for example, includes both intra-organismal DNA of microorganisms and extra-organismal DNA derived from the faeces and mucus of macro-organisms in environmental media (eDNA *sensu lato*).^4^

This approach, which entails the direct extraction of DNA from environmental media to study the distribution of organisms, initially began with studies on bacteria and other microorganisms,^5^ the techniques of which made it possible to determine the types of bacteria present in soil or river water without the necessity of culturing specimens. These methods of extracting DNA directly from environmental media and its subsequent analysis are now commonly used as tools for characterizing the bacterial or fungal flora within a given environment.^6^^,^^7^ In contrast, eDNA analysis of macro-organisms is still at a comparatively nascent stage. It was in 2008, in their study on bullfrogs, that Ficetola et al.^8^ were the first to demonstrate that eDNA could be employed to determine the distribution of macro-organisms. Since then, along with the ongoing advances in biotechnology, the use of extra-organismal DNA to investigate the distribution of macro-organisms has undergone a notably rapid development. In this article, I mainly summarize the current status of eDNA analysis for macro-organisms (organisms that can be visualized without a microscope, unlike microorganisms), and further discuss prospects regarding methods used to obtain information on macro-organisms based on analyses of nucleic acids derived from environmental sources, using new techniques based on molecular biology and molecular genomics.

## Properties of eDNA

That which we referred to as the eDNA of macro-organisms in water includes the following states: (i) naked DNA (dissolved DNA): DNA that has been released from cells and exists in a dissolved state in water; (ii) particle-bound DNA: DNA bound to particulate material, such as small mineral particles; (iii) intra-membrane DNA: DNA contained within cell organelles, intact cells, or parts of tissues. With respect to macro-organism-derived eDNA, particles trapped by filtration through filter paper with a mesh size of 0.45–1.5 μm are often used for analysis,^9^ and consequently, the main targets of analyses are (ii) or (iii). Indeed, the majority of carp eDNA detected has been isolated from particles larger than 0.2 μm, and thus analysing particle-bound and intra-membrane DNA represents the most productive approach.^10^ Although the nature of particle-bound DNA is not well understood, in an experiment in which naked DNA was added to field water, the majority was trapped by filtration,^11^ and thus particle-bound DNA also makes up a certain proportion of the eDNA analysed. In some cases, intracellular organelles or cells may be present in water, as is discussed below.

Although analyses of macro-organism-derived eDNA typically target mitochondrial DNA for animals or chloroplast DNA for macroalgae and macro-plants, nuclear DNA is occasionally assessed.^12^ This preference for mitochondrial DNA and chloroplast DNA is based on the fact that they occur as multiple copies within a single cell, which is advantageous in terms of detection, as is the availability of considerable amounts of mitochondrial and chloroplast DNA sequence information in the international nucleotide sequence databases (INSDs) such as DNA Data Bank of Japan (DDBJ), European Nucleotide Archive (ENA), and GenBank.

The origin of eDNA and the processes, by which eDNA released from organisms attains the aforementioned three states, are a particular focus of research; however, although a number of studies are ongoing, we currently lack a complete understanding. Taking fish as an example, the possible origins of eDNA include faeces, body surface mucus, sperm, eggs, and carcasses. However, there has been insufficient research to determine the proportional contributions of each of these sources to total eDNA.

Among the fundamental properties of eDNA, the most well studied is the process of degradation. In this regard, numerous studies have reported that eDNA undergoes exponential decay,^13^^,^^14^ although the determined rate of degradation has been found to differ significantly among studies, which is plausibly attributable to a range of causal factors, including bacterial abundance, water temperature, pH, and other biotic and abiotic factors. In addition, there have been a number of modelling studies, such as those applying biphasic and Weibull models, which assume more complex degradation processes, and have greater explanatory power.^15–17^ Although the specific mechanisms remain to be established, degradation is assumed to be a multi-step process. For example, intracellular DNA may initially undergo conversion to intra-organelle DNA and is thereafter transformed to naked or particle-bound DNA via a different process, thereby leading to a complex degradation pattern, with different enzymes being involved at each of the different stages. It has also been reported that nuclear and mitochondrial DNA are characterized by differing rates of degradation,^18^ although the underlying mechanisms remain to be clarified.

The distribution of eDNA reflects the spatial distribution of its ‘owners’, and a number of studies have been conducted on the distribution range of extra-organismal DNA, albeit with contrasting findings. For example, studies on the downstream distribution of eDNA have tended to yield wide-ranging estimates from <100 m^19^ to >10 km.^20^ Such disparities could conceivably reflect differences in the state of eDNA depending on target species, differences in initial concentration depending on the population and biomass of organisms, and differences in transportability in different rivers. With regards to fish, numerous studies have shown that even in the case of species with relatively dense populations in certain areas, DNA is detectable within 1–2 km,^21^^,^^22^ whereas in marine environments, distances of within a few hundred meters have been reported.^23^^,^^24^

## Analytical strategies for macro-organism eDNA

Since the publication in 2008 of the first paper describing estimates of organism distribution based on analyses of macro-organism-derived eDNA,^8^ the feasibility of surveying the distribution of different groups in a range of aquatic ecosystems has become well established. There are essentially two types of analytical strategies, namely, species-specific detection of eDNA (also referred to as ‘eDNA barcoding’) and exhaustive detection of the eDNA of certain taxa (referred to as ‘eDNA metabarcoding’). Both have their respective advantages and disadvantages and accordingly should be used depending on need (Table 1).

**Table 1:** The strengths and weaknesses of species-specific and exhaustive eDNA analyses

Analytical methods	Strengths	Weaknesses
Species-specific analysis (eDNA barcoding)	Simple and inexpensive compared with metabarcoding	Assays need to be developed for each target
	Higher sensitivity than metabarcoding Compatible with quantitative methods	Only single species information available in a single analysis
Exhaustive analysis (eDNA metabarcoding)	Multispecies information available in a single analysis	Labour intensive and costly
	Assays available for a variety of purposes	Not highly quantitative

## Species-specific eDNA detection and quantification

Species-specific eDNA analysis (eDNA barcoding) is an analytical method that focuses on a single species of interest. In the first documented example, Ficetola et al.^8^ reported the feasibility of investigating the distribution of larval bullfrog (*Rana catesbeiana*), an invasive species in Europe. Such methods are particularly useful for studying the distribution of rare or exotic species of interest, and have been used extensively, particularly in many of the early studies. Examples include the detection of endangered salmonids in US rivers^25^ and the distribution of the invasive bluegill sunfish in Japanese ponds.^26^ In some applications, a rare species and closely related invasive alien species can be simultaneously detected from a single sample.^27^

The advantages of species-specific analysis are that it is simple, inexpensive, and relatively easy to prevent contamination. Most simply, the presence or absence of target species DNA can be determined by the presence or absence of an expected band using conventional polymerase chain reaction (PCR) and subsequent electrophoresis. In addition, as described below, this approach is compatible with quantitative methods, and it is relatively straightforward to determine the concentration of eDNA using real-time or digital PCR.^28^^,^^29^ Conversely, however, among its weaknesses is that it is necessary to develop a specific detection assay for each target species. Typically, the development of such assays for eDNA analysis necessitates the design of primers using database information, followed by *in silico* confirmation and *in vitro* testing to ensure assay specificity. If, however, the database information proves insufficient, it is then necessary to determine the DNA sequences of the target species and its sympatric relative. Predictably, these processes are both time-consuming and costly, and when multiple species are targeted, these procedures need to be repeated for each target species. Having established these assays, however, the subsequent operations are relatively straightforward and inexpensive.

As previously mentioned, it is possible to determine DNA concentrations for a given species in the environment using quantitative PCR methods. Positive correlations between eDNA concentrations in a particular water body and the abundance or biomass of organisms have been reported from the earliest days of macro-organism eDNA studies, not only in closed ecosystems such as lakes and ponds but also in open ecosystems such as lotic water bodies and oceans.^23^^,^^28^^,^^30–32^ However, whereas relative quantification of abundance or biomass is eminently feasible, seasonality in the concentrations or detection frequencies of eDNA^33^^,^^34^ tend to make the absolute quantification of abundance and biomass more challenging. Among related studies, the most successful example of a large-scale absolute abundance estimation is that in which Fukaya et al.^35^ quantified the abundance of Japanese jack mackerel (*Trachurus japonicus*) in the ocean. In this study, the numbers of Japanese jack mackerel in a bay estimated based on eDNA quantification and by echo sounder were almost identical, thereby demonstrating the feasibility of quantifying the absolute number of fish individuals using eDNA. However, the execution of this study required considerable effort, including modelling the flow field in the bay, using parameters of eDNA release and degradation rates obtained from tank experiments, as well as selecting a suitable season and an area with no appreciable variation in the size of the target fish species. This study accordingly highlights that whereas absolute abundance estimates based on eDNA quantification are achievable, we have yet to reach the stage at which we can readily determine the population density of a particular fish species by simply sampling water.

## Exhaustive detection of certain taxa

Exhaustive eDNA detection (eDNA metabarcoding) is a method that can be applied to comprehensively identify species in a certain ecosystem using universal primers to amplify the DNA of species belonging to a specific taxon, and then successively analysing the amplified sequences. This method, which at the earliest stages of development necessitated labour-intensive manual sequence reading,^36^ was subsequently combined with high-throughput sequencing (HTS; also referred to as next-generation sequencing)^30^^,^^36^ and has become a major tool in eDNA analysis. Using this technique, it has been reported that several dozen to more than 100 species of fish DNA can be detected in as little as a litre of water.^37^

The most advanced eDNA metabarcoding studies on macro-organisms conducted to date have been those performed for fish, for which a number of different gene regions have been used as markers for successful metabarcoding. Whereas early universal primers targeted CytB,^30^^,^^36^ more recently, primers targeting rRNA coding regions, such as 12S rRNA and 16S rRNA, have become mainstream.^37–39^ When designing such primers for metabarcoding, it is necessary to strike a balance between conservativism and specificity in primer regions and the length and taxonomic resolution of the amplified region (the internal region between the primers). With respect to fish, the MiFish primer, which targets 12S rRNA, provides an excellent balance in this regard and is used worldwide.^40^ By employing this primer, it is now possible to study the diversity of fish species in any aquatic ecosystem, including freshwater, brackish water, coral reefs, and the deep sea.

Although apart from fish, comparatively few eDNA metabarcoding studies have been conducted for other taxa, in the case of amphibians, batra, a universal primer set for 12S rRNA, has been reported to significantly increase detection probability compared with sampling surveys,^37^ whereas the universal primer set for 16S rRNA (Amp16S) has been reported to detect the presence of a greater variety of species than physical surveys.^41^

There have also been reports of the use of eDNA metabarcoding for the detection of terrestrial taxa,^42^^,^^43^ an example of which is the detection of DNA derived from mammals visiting natural saltlicks in Borneo.^44^ With respect to invertebrates, successful metabarcoding has been reported for decapods^45^ and in the Anthozoa.^46^^,^^47^ Moreover, there are also examples of studies that have been performed for broad taxonomic groups, including the entire group of eukaryotes.^48^ It is widely anticipated that in the near future metabarcoding assays will be developed for a range of taxonomic groups, thereby making it possible to detect any species in any water body, simply by sampling a bucketful of water.

Although compared with quantitative PCR, metabarcoding is a less quantitative technique, efforts have been made to semi-quantify the analysis of eDNA in water. Ushio et al.,^49^ for example, have developed a method to simultaneously quantify the copy number of eDNA from different species using internal standards, demonstrating that it is possible to quantify DNA from more than 70 species in samples of field water. Furthermore, despite being considered less reliable than qPCR in terms of quantitative accuracy, Hoshino et al.^50^ showed that absolute quantification is possible via quantitative sequencing based on the addition of random tags. As these techniques become more advanced, it will undoubtedly become possible to quantify multiple species using eDNA (by metabarcoding), which would be particularly beneficial from the perspective of characterizing the dynamics of biological communities.

## Analysis of intraspecific variation

Whereas the eDNA analysis described in the preceding section is primarily designed to achieve species-level resolution, if the resolution of analysis can be further enhanced, it would conceivably be possible to detect intraspecific polymorphisms, such as differences between local populations. In this regard, Uchii et al.^51^ have demonstrated the potential of cycling probe technology, which can detect single-nucleotide polymorphisms, for quantitatively distinguishing between mitochondrial genes derived from native and non-native populations of common carp (*Cyprinus carpio*) in Japan.

A further example of higher resolution eDNA analysis is the detection of intraspecific polymorphisms using HTS targeting the rapidly evolving D-loop region of mitochondria, as exemplified by the successful detection of intraspecific polymorphisms in the ayu (*Plecoglossus altivelis*) from aquarium and natural river water.^52^^,^^53^ However, the detection of intraspecific polymorphisms based on HTS can be affected by noise due to PCR errors^54^ and sequencing errors.^55^ Consequently, necessary precautionary measures should be taken to reduce such errors by using low-error enzymes and conservative denoising in data analysis.

The aforementioned methods used for detecting intraspecific polymorphisms based on eDNA analyses typically use relatively short marker regions, single-nucleotide polymorphisms, or rapidly evolving regions, which are well suited to eDNA analysis. In recent years, however, it has been demonstrated that considerably longer gene fragments are present in environmental media. For example, Kakehashi et al.^56^ amplified gene fragments of several thousand base pairs in length from eDNA derived from aquarium water and successfully combined these to determine almost the entire length of mitochondria. In a further study, Deiner et al.^57^ showed that virtually complete mitochondrial genomes can be successfully amplified from environmental water using long-range PCR. Although these methods are currently at the proof-of-concept stage, if they can be successfully implemented in practical use, it will be possible to perform analyses that require higher resolution, thereby substantially expanding the scope of eDNA analysis.

## The reconstruction of ancient biota

Analysis of macro-organism-derived eDNA also offers the prospect of reconstructing past biota, and since the early 2000s, efforts have been made to extract DNA directly from sediment or permafrost cores, as opposed to fossils, frozen bodies, or other obvious biological remains. Indeed, DNA extracted from Siberian permafrost core samples has been found to contain mammoth DNA from between 20,000 and 30,000 yrs ago and plant DNA dating back to between 300,000 and 400,000 yrs ago.^58^ Similarly, attempts have been made to determine how fauna and flora have changed over time based on analyses of sediment cores collected from the bottom of lakes. For example, in a study conducted on sediment obtained from an Alpine lake, Giguet-Covex et al. sought to examine how vegetation growing in the vicinity of the lake has changed over the past several thousand years, and also to identify the types of animals that have inhabited the lake surroundings. Their findings thus enabled these authors to document how livestock farming has developed in this area.^59^

Reconstructions of the aquatic fauna from the more recent past using sediments from water bodies are also in progress, among which studies have measured variations in DNA abundance of specific fish species dating back decades to centuries.^60^^,^^61^ Although conducting eDNA metabarcoding on such samples has the potential to reveal changes that have occurred in the assemblages of aquatic fauna, the success of these methods has to date been limited to particular environments in which eDNA degradation is exceptionally slow, such as high-altitude lakes or oxygen-deficient deep-sea areas. Nevertheless, the more widespread application of these techniques could provide indices for environmental restoration.

## The weaknesses of current eDNA analyses

Given its considerable advantages, eDNA analysis is rapidly emerging as a practical method for monitoring the distribution of organisms in the field, particularly in aquatic environments. For example, in the European Union, UK, USA, Japan, and elsewhere, this approach is routinely used in governmental biomonitoring programmes^62–65^ and is destined to become the golden standard for biological monitoring. Nevertheless, despite the multiple advantages of eDNA analysis, the use of this technique is currently constrained by certain weaknesses compared with traditional survey methods.

The first of these drawbacks is a lack of information regarding the status of detected organisms, such as the size and sexual maturity of individuals. In the case of catch-based surveys, it is possible to directly obtain information as to whether the captured individuals are adults or juveniles, as well as their sexual maturity and nutritional status. Such information can serve as vital indicators with respect to population health, and also provide relevant details for ecosystem conservation. In this context, determining whether invasive species are reproducing is of particular importance with respect to undertaking the necessary measures to control these alien species. Consequently, the lack of such information on organism status is considered perhaps the most fundamental of the weaknesses of current methods of eDNA analysis.

A second major deficiency of eDNA analysis is the inability to detect the occurrence of hybridization. For example, if native species and closely related exotic species occur sympatrically, it is of particular conservation importance to determine whether these are hybridizing. However, using the current methods of eDNA analysis, even if the DNA of both native and exotic species are detected simultaneously,^27^ it is still not possible to ascertain whether they are simply inhabiting sympatrically or actively hybridizing.

The following sections discuss the future of eDNA research, including expectations for new technologies and resources to overcome these weaknesses.

## The future of eDNA research

**Figure 1:**
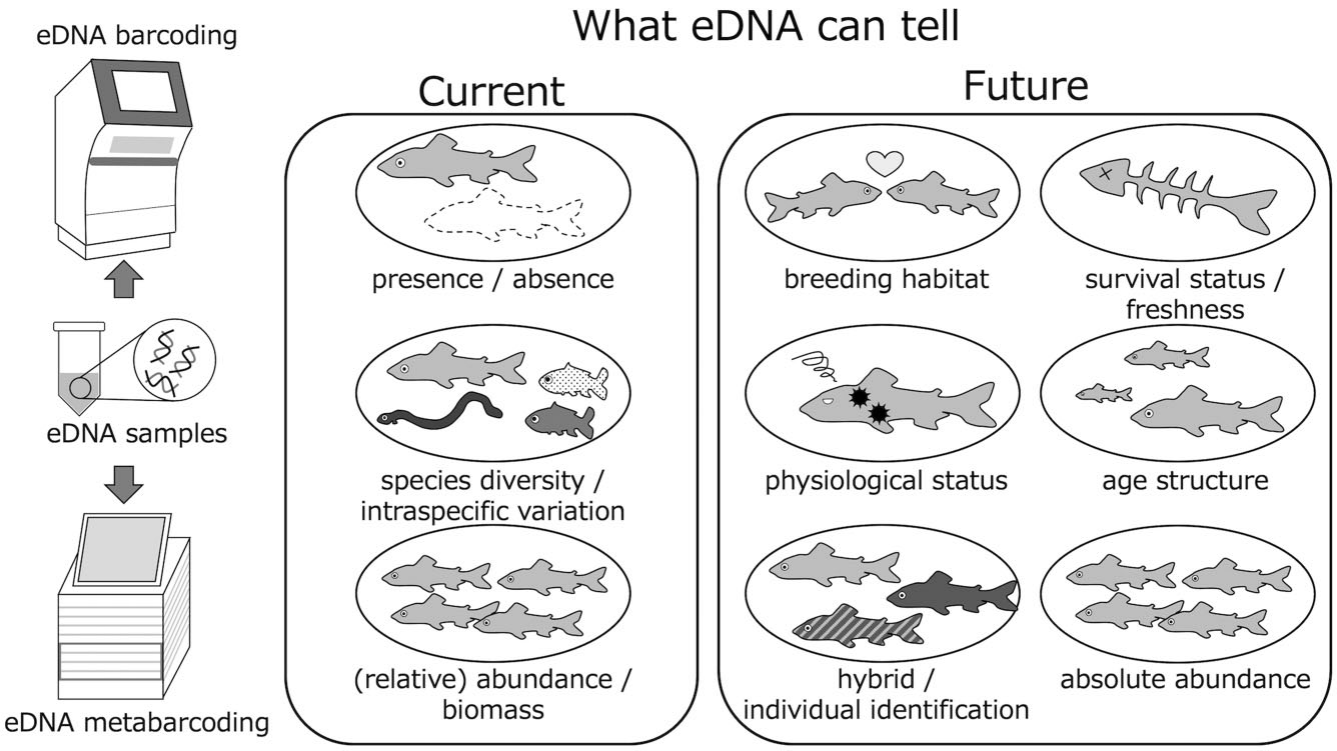
Overview of current eDNA analysis and future perspectives. Currently, eDNA analysis is focused mainly on species distribution and relative abundance or biomass. Information on behaviour, physiology, age structure, and other aspects of the organism’s condition that cannot be monitored with current technologies may be obtained in the future.

As previously indicated, eDNA analysis can be used to determine the current and past distributions of organisms. More recently, however, researchers have begun to focus on expanding the scope of eDNA beyond merely determining the presence or absence of species, as highlighted by developments envisaged by the eDNA Society, which set the theme of its 2021 annual meeting as ‘Species distributions, and beyond’.^66^ The following sections describe efforts designed to extract information other than species distributions from environmental media by combining established and new technologies, including nuclear eDNA, longer fragment, eDNA epigenomics, environmental RNA (eRNA), and environmental single-cell analyses (Fig. 1; Table 2).

**Table 2:** Future perspectives of eDNA analysis and related technologies

Expected new technologies	Possible applications
Nuclear eDNA analysis	Identification of breeding habitat
Longer fragment analysis	Determination of survival status
	Estimation of the freshness of eDNA
eRNA analysis/eDNA epigenomics	Determination of physiological status
	Identification of age structure
Environmental single-cell analysis	Determination of physiological status
	Detection of hybrid individuals
	Identification of individuals
	Estimation of abundance

## Identifying breeding habitats

Of particular importance with respect to the conservation of rare species and the control of invasive species, is not only whether these species are present but also whether they are breeding and the locations of their breeding habitats. Initiatives that aim to determine breeding grounds based on eDNA analysis have been ongoing for a number of years, most simply, by determining habitats during the known breeding season.^67^ However, in recent years, efforts have been made to identify breeding sites and seasons with higher precision by detecting specific signals associated with the breeding behaviour of target species.

As externally fertilized animals, most fish species release large volumes of sperm into the environment during the breeding season, and given that sperm is among the sources of eDNA, it is predictable that the concentrations of such eDNA will increase during the breeding season. Examples include the hellbender (*Cryptobranchus alleganiensis*), the eDNA levels of which have been reported to increase in breeding habitats during the breeding season.^68^^,^^69^ However, it is well known that the rate of eDNA release is temperature dependent,^70^ with an associated seasonality in eDNA concentration or detection sensitivity.^33^^,^^34^^,^^71^ In addition, concentrations vary naturally with the distance from individuals releasing the eDNA. Consequently, there are limitations to estimating reproduction based solely on simple changes in eDNA concentration.

Recently, considerable attention has focused on detecting breeding behaviour using the ratio of nuclear to mitochondrial eDNA, which is based on the low number of mitochondria in sperm. For example, in the case of the Macquarie perch, Bylemans et al.^72^ found that experimental sperm input increased the nuclear/mitochondrial eDNA ratio in an aquarium. Moreover, these authors also reported that the nuclear/mitochondrial eDNA ratio increases during the breeding season in the field. This technique is accordingly expected to contribute to the identification of the breeding sites and seasons of wild animals. However, such efforts are still at a comparatively nascent stage, and the applicability of this method in different ecosystems and for different species needs to be further assessed. For example, aquarium experiments conducted in the author's laboratory have revealed that in some cases, the nuclear/mitochondrial eDNA ratio declines during the breeding season of sea cucumbers, which may reflect the low nuclear/mitochondrial DNA ratio in eggs. The nuclear/mitochondrial eDNA ratios during the breeding season may differ depending on the ecology of the target species, the shape of gametes, and their environmental dispersion, all of which need to be further investigated.

## Determining survival status

Techniques that use multiple markers to obtain information beyond the distribution of organisms may be useful not only for understanding reproductive behaviour but also for understanding the survival status of the source organisms. One criticism of eDNA analysis is that contamination with DNA derived from dead organisms and/or their products can skew results. For example, eDNA derived from food discharged via sewage, fish-consumed bird droppings, or the corpses of deceased individuals, can result in the detection of eDNA even in the absence of live target species.^73^^,^^74^ This is exemplified by the findings of a study conducted by Yamamoto et al.,^23^ who quantified the eDNA of Japanese jack mackerel in a bay and compared the findings with estimates based on echo sounder detection. However, whereas extremely high concentrations of eDNA were detected near a fishing port, no fish were identified near the port by an echo sounder. The authors assumed this disparity to be attributable to the presence of a nearby fish market, contamination from which influenced the distribution of eDNA in the bay. To address this type of problem, Jo et al.^75^ focused on the process of eDNA degradation. eDNA is assumed to fragment over time, and the authors thus hypothesized that the use of longer eDNA markers would contribute to minimizing the likelihood of detecting DNA derived from dead fish. This prediction was proved correct, with the authors successfully eliminating the influence of market-derived DNA using the same sample examined by Yamamoto et al.^23^

As longer genetic markers have a shorter detectable time than shorter markers, the application of this principle could yield potentially important insights regarding the time scale represented by eDNA. In addition, based on the assumption that immediately after release, DNA should be long, there would theoretically be no difference in the copy numbers between long and short markers at this time, whereas with the passage of time following its release, eDNA is expected to have a progressively lower long/short eDNA copy ratio. Accordingly, this approach would enable the incorporation of a time axis in eDNA analysis. Similarly, it has been confirmed that nuclear DNA and mitochondrial DNA have different degradation rates,^18^ and consequently, by using a combination of multiple markers with different rates of degradation, it may be possible to estimate the time that has elapsed since detected DNA was released (i.e. the freshness of eDNA).

## Organism status

If the physiological status of organisms can be determined based on analytical methods using eDNA or similar materials, it may become feasible to conduct ‘health checks’ on wildlife. To determine whether organisms are under stress, sick, or in good nutritional health by using water samples, this would contribute not only to conserving ecosystems but could also be applied in other fields, including aquaculture.

One novel approach that might potentially be applied in assessing the physiological status of organisms is the analysis of eRNA, which in contrast to DNA that remains essentially unchanged from birth to death, can undergo changes in expression depending on the stage of growth and physiological state of an organism. Consequently, if, for example, the relative abundance ratio of genes that are specifically expressed when an organism is stressed is high, it might be possible to determine whether that organism is under stress. Such an analysis may be challenging to perform on natural populations but could be useful in aquaculture farms that house single species. Similarly, if a gene specifically expressed at the larval stage can be detected, the presence of larvae could accordingly be verified. Although, given that RNAs are more unstable than DNA, a technological breakthrough might be necessary to enable the routine detection of RNA in the environment, on the basis of tank experiments, Tsuri et al.^76^ succeeded in detecting mRNA of organ-specifically expressed genes in zebrafish. Similarly, Miyata et al.^77^ have successfully performed the eRNA metabarcoding of fish by targeting the mitochondrial 12S rRNA gene from field water samples. These findings indicate that eRNA analysis of macro-organisms, including fish, is eminently feasible. Accordingly, using eRNA to detect and quantify functional genes from field samples could well facilitate the development of methods that enable elucidation of the growth stages and physiological states of organisms.

## eDNA epigenomics

Whereas the sequence of genomic DNA remains essentially unchanged from birth to death, the state of DNA modification, such as methylation, undergoes constant change. It is thus conceivable that such epigenomic changes could also be used to assess the status of organisms. For example, the methylation and demethylation of cytosines have been shown to be associated with the suppression and activation of gene expression, respectively.^78^ Consequently, if we can determine the methylation status of a specific gene, it may be possible to establish the state of an organism, similar to eRNA analysis that uses the expression level of functional genes as an indicator. Moreover, given that the methylation state of DNA at a particular residue can be quantified by bisulphite sequencing or the use of methylation-sensitive restriction enzymes,^79^^,^^80^ such techniques may also have application in eDNA analysis.

In addition to assessments of organism status, methylation-related analyses may also enable age structure determination. In zebrafish, for example, it has been established that genome-wide methylation rates change with age,^81^ which thus indicates the feasibility of determining the average age composition of individuals in a given water body. Such information would be of particular importance with respect to the conservation of long-lived organisms. For example, whereas the Japanese giant salamander (*Andrias japonicus*) is believed to live for more than 100 yrs, a decline in the number of young individuals has been noted in recent years.^82^ This would accordingly indicate that the population is currently ‘ageing’, and thus may be unsustainable in the long term. However, determining age structures using conventional methods is notoriously difficult, and thus employing eDNA analysis to determine age structures could provide an effective means of understanding the sustainability of populations.

## Environmental single-cell analysis

Earlier it was mentioned that the full length of the mitogenome is detectable in environmental water, and size fractionation revealed an abundance of eDNA larger than 10 μm in size.^18^ Given these findings and taking into consideration that vertebrate cells are ∼10–20 μm in size, it is believed highly likely that there are single vertebrate-derived cells widely distributed in the environment.

Recent technological advances have led to the rapid development of single-cell-based analysis methods, such as single-cell RNA-seq,^83^ and thus, if we could collect individual ‘environmental single cells’ of different species in certain environments, it might well be feasible to determine the various physiological states of their ‘owners’. Theoretically, using this approach it would be possible to simultaneously analyse the physiological states and growth stages of different species living in a given area. Moreover, analysis at the cellular level could open up the possibility of identifying individuals and determining hybrids, which have hitherto proved difficult using current eDNA techniques. Furthermore, given the feasibility of individual identification, it might then be possible to estimate the number of individuals using methods analogous to mark-recapture-based surveys.

## Conclusion

In this review, I have touched upon the current status and future developments of eDNA analysis and related methods. eDNA-based monitoring has already become established as a ground-breaking novel technology, with significant implications for fields such as ecology. These developments have and continue to be made possible by techniques that have been developed in the fields of molecular biology and molecular genomics. It is anticipated that further advances will be founded on the integration of cutting-edge technologies that include more accurate long-read metabarcoding and environmental single-cell analysis. To date, microbiology and macrobiology have tended to develop independently with comparatively little cross-fertilization; however, technology-mediated fusion has the potential to bridge the gap between these discrete disciplines and thereby contribute to the development of an integrated biology.
